# Feasibility of implementing time‐restricted eating in women with mild cognitive impairment

**DOI:** 10.1002/alz.089509

**Published:** 2025-01-03

**Authors:** Matthew Thomas, Gregory A Jicha, Jordan P. Harp, Philip A Kern, Dorothy D Sears, Dara L James, Yuriko Katsumata, Julie S Pendergast

**Affiliations:** ^1^ University of Kentucky, Lexington, KY USA; ^2^ University of Kentucky Sanders‐Brown Center on Aging, Lexington, KY USA; ^3^ University of Kentucky / Sanders‐Brown Center on Aging, Lexington, KY USA; ^4^ Arizona State University, Phoenix, AZ USA; ^5^ Sanders‐Brown Center on Aging, University of Kentucky, Lexington, KY USA

## Abstract

**Background:**

Disruption of sleep and circadian rhythms are associated with cognitive decline, preclinical Alzheimer’s Disease (AD) pathology, and increased risk of dementia. Alleviating circadian rhythm and sleep disruption may improve cognition and reduce the progression of AD and related dementias (ADRD). Time‐restricted eating (TRE), a circadian behavioral intervention that corrects disrupted eating rhythms by aligning food intake to the daytime, has demonstrated improvements in metabolic dysfunction and sleep quality. Individuals who adhere to TRE are less likely to have cognitive impairment compared to those who consume meals with no time restrictions. To date, no studies have investigated the potential efficacy of a TRE intervention to improve cognition among individuals living with cognitive impairment. This study will determine the feasibility of TRE in women with mild cognitive impairment (MCI).

**Method:**

Women diagnosed with MCI will be recruited from the University of Kentucky Sanders‐Brown Center on Aging cohort to participate in a pre‐post study. Data will be collected during a 2‐week baseline period and an 8‐week TRE intervention period. During baseline, measures of cognition, food timing, activity/sleep, and metabolic parameters will be collected. For the duration of the enrollment, first and last calorie intake times will be collected from participants with an SMS texting system. Participants with mean daily calorie durations of at least 12 hours and calorie intake after 8:00pm on the majority of nights during baseline will be eligible for the TRE intervention. During the TRE intervention, each participant will self‐select a 10‐h window (ending by 8:00pm) during which they will consume all daily calories during the 8‐week TRE intervention. At the end of the intervention, baseline measures will be repeated. Adherence to the TRE protocol is the primary outcome of this study.

**Result:**

We anticipate enrolling 22 participants in a screening, baseline phase to enroll 15 eligible participants for the TRE intervention.

**Conclusion:**

This pilot study will provide insight into the feasibility of conducting a TRE intervention among women living with MCI and provide preliminary data to calculate sample size for a future randomized controlled trial testing the efficacy of TRE in improving, or preventing further decline, in cognition.

